# Molecular Context of ADAR-Mediated Editing of Coding RNA in Colorectal and Lung Cancers

**DOI:** 10.3390/ijms27062625

**Published:** 2026-03-13

**Authors:** Alexander Modestov, Daniil Luppov, Ivan Gaziev, Nikita Golushko, Galina Zakharova, Marianna Zolotovskaia, Elena Poddubskaya, Alexander Seryakov, Vladimir Prassolov, Marina Sekacheva, Anton Buzdin

**Affiliations:** 1 Institute for Personalized Oncology, Biomedical Science & Technology Park, Sechenov First Moscow State Medical University, 119991 Moscow, Russia; modestov_a_a@staff.sechenov.ru (A.M.);; 2 Scientific Center of Genetics and Life Sciences, Sirius University of Science and Technology, 354340 Sirius Federal Territory, Russia; 3 Vitamed Oncological Clinical Center, 121309 Moscow, Russia; 4 Medical Holding SM-Clinic, 105120 Moscow, Russia; 5 Engelhardt Institute of Molecular Biology, 119991 Moscow, Russia

**Keywords:** ADAR-mediated RNA editing, ADAR1, ADAR2, RNA sequencing, lung cancer, colorectal cancer, transposable elements

## Abstract

RNA editing is a critical post-transcriptional modification that contributes to transcriptomic and proteomic diversity. The most common A-to-I (recognized as G) RNA editing enzymes are adenosine deaminases acting on RNA 1 and 2 (ADAR1 and ADAR2, respectively), which mediate alterations across all regions of mRNA molecules. However, a systematic cross-tissue view of RNA editing and its molecular correlates is still lacking. Here, we developed a rapid method for ADAR editing assessment based on 24 frequently edited positions in coding regions, which enables faster estimation of RNA editing levels than previous methods. We applied this metric to assess RNA editing in normal and cancerous lung and colorectal tissues. We analyzed RNA and whole exome sequencing profiles of experimental 172 colorectal and 144 lung cancer samples, and literature 646 colorectal and 1037 lung cancer samples. We also examined two types of control tissues: tumor-matched normal tissues (51 colorectal and 108 lung samples) and healthy tissues (6 colorectal and 7 lung samples). Overall ADAR-mediated RNA editing levels were ~2.9- and ~4.7-fold higher in healthy controls than in colorectal and lung cancers, respectively. In addition to their well-known association with immune cells, we identified positive correlations of ADAR editing with 740 molecular pathways including those responsible for extracellular matrix organization, RAS-MAPK axis and G2/M phase cell cycle arrest, and negative—with 139 pathways responsible for DNA repair, apoptosis, expression of transposable elements, and other factors.

## 1. Introduction

ADAR-catalyzed deamination of adenosine (A) to inosine (I) represents the predominant type of RNA editing in mammals [1,2,3,4]. This A-to-I modification takes place within double-stranded RNA (dsRNA) hairpins formed either in mRNAs or in various classes of non-coding RNAs. In precursor mRNAs, the substrates of ADARs typically consist of imperfectly paired duplex regions generated by complementarities between exonic segments and adjacent intronic or regulatory elements, including untranslated regions (UTRs). These dsRNA structures are selectively bound by ADAR proteins through their RNA recognition domains [2,4,5]. Following editing, the intronic sequences together with the dsRNA hairpins are eliminated during splicing [3]. Because cellular decoding machinery interprets inosine as guanosine, A-to-I conversions may introduce nonsynonymous substitutions or give rise to novel splice junctions. Although many edited hairpins lie outside coding regions and persist in mature transcripts as components of the 3′-UTR, where they regulate translation efficiency and global mRNA stability [6], ADARs additionally influence the biogenesis and target selection of non-coding RNAs such as microRNAs and circular RNAs [7,8,9]. However, the most functionally consequential events are A-to-I edits occurring within protein-coding regions.

The ADAR protein family comprises three enzymes—ADAR2 (ADARB1), ADAR3 (ADARB2), and ADAR1 produced in two isoforms, p150 and p110. These two isoforms have distinct functional impacts, where p150 is primarily located in the cytoplasm and is crucial for innate immunity and germinal center response during B cell maturation. In turn, p110 is involved in general housekeeping editing and has primarily nuclear localization [10,11,12]. All these proteins share a similar modular organization characterized by a conserved C-terminal deaminase domain and multiple dsRNA–binding domains (three such domains in ADAR1, and two in ADAR2 and ADAR3) [13]. Additional structural features distinguish individual family members: ADAR1 possesses Z-DNA–binding domains, whereas ADAR3 contains an N-terminal arginine-rich region (R-domain) that preferentially associates with single-stranded RNA [4,14]. The two ADAR1 isoforms arise from distinct promoters: the p110 variant is produced from a constitutively active promoter, while the p150 isoform is transcribed from an interferon-responsive promoter and participates in modulating interferon signaling and innate immune defenses against foreign RNAs [10,15,16].

ADAR1 functions as a negative regulator of interferon signaling and modulates innate immune defenses against exogenous dsRNAs [17]. This process has a central role in the maturation of B cells and in preventing the autoimmunity [10].

A central aspect of its activity is the editing of endogenous dsRNA structures, which prevents these molecules from being misidentified as viral RNA species that would otherwise trigger the RIG-I-like receptor cascade and induce type I interferon production. Beyond this antiviral safeguard, ADAR1 has been shown to modify a variety of cellular transcripts implicated in distinct molecular pathways [18,19,20]. Alterations in ADAR1 abundance or domain composition can contribute to pathological states; for example, dysregulated *ADAR1* expression can promote aberrant interferon production and potentiate autoimmune responses [10,15,16]. Furthermore, recent evidence indicates that the p110 isoform of ADAR1 participates in the resolution of R-loops at telomeric repeats by editing RNA within RNA:DNA hybrid structures, thereby helping maintain genomic stability in tumor cells [21].

*ADAR2* is expressed at particularly high levels in the central nervous system, where it predominantly carries out precise A-to-I edits within short RNA hairpins [22]. A well-characterized substrate of ADAR2 is the *GRIA2* gene mRNA, which encodes the B subunit of the AMPA-type glutamate receptor. Editing of this transcript causes glutamine to arginine substitution, thereby producing an AMPA receptor channel variant that restricts Ca^2+^ entry—a modification essential for proper neuronal physiology [23]. Diminished ADAR2 activity, and consequently insufficient editing of *GRIA2* has been associated with impaired motor neuron function in amyotrophic lateral sclerosis [4].

Growing evidence indicates that RNA editing activity, expression of ADARs, and particular edited sites may serve as prognostic biomarkers of cancer progression, as dysregulated editing can result in the accumulation of cells with multiple altered proteins, thereby promoting tumor heterogeneity and adaptability [14,24]. In most studies, RNA editing events have been analyzed using RNA sequencing data from matched normal tissues of the same patients [2,25]. In certain studies, significant differences in editing activity were identified between matched tumor and normal samples: over-editing was detected for head and neck, breast, and thyroid cancers, whereas kidney cancers showed predominant under-editing of some particular positions within mRNA. However, for the majority of editing sites, tumor and normal tissues displayed similar editing levels [25]. At the same time, several studies have reported lower *ADAR2* levels in tumors compared with normal tissues, along with an opposite trend for *ADAR1* [12,26,27].

Aberrant expression patterns of ADARs—both across distinct tumor types and within individual cancer entities when compared with normal tissues—can help delineate tumor-specific molecular signatures, thereby improving our understanding of malignant progression and facilitating the development of more effective therapeutic approaches [28]. This issue is particularly important because RNA editing in tumors may occur within coding regions, ultimately expanding proteomic diversity during oncogenesis [29]. In this context, it is essential to account for RNA editing mediated by both ADAR1 and ADAR2 in tumor tissues, since although both enzymes are capable of editing dsRNAs, ADAR2 preferentially targets coding regions, whereas ADAR1 predominantly edits Alu regions [14,28,30,31]. Accordingly, global RNA editing activity has been assessed using various approaches based on the analysis of all known A-to-I sites, Alu regions, or sites resulting in amino acid substitutions. Because the most functionally consequential events are A-to-I edits occurring within protein-coding regions, we compared existing approaches and developed a novel method that captures the activity of both ADAR1 and ADAR2.

In this study, we introduce a new metric for rapid quantification of RNA editing activity based on the high-confidence positions of coding regions detectable in RNA-seq datasets. In addition, we performed a comprehensive correlation analysis linking editing levels with molecular pathways and activities of transposable elements.

## 2. Results

### 2.1. Comparison of Metrics for RNA Editing Quantification

To characterize global RNA editing activity, we compared the proposed metrics, weighted RNA editing and mean RNA editing, with several established approaches that estimate overall editing levels by averaging editing signals across predefined sets of sites. Specifically, we applied the overall editing level (OverallIndex), the recoding editing index (RecodingIndex, REI), and the Alu editing index (AEI). The OverallIndex summarizes the proportion of edited reads across all known A-to-I editing sites annotated in REDIportal, whereas the RecodingIndex focuses on a curated subset of recoding sites that result in nonsynonymous substitutions in protein-coding genes. In contrast, the AEI captures global RNA editing activity within repetitive elements by quantifying coverage-weighted editing levels across Alu elements. All five metrics were calculated for the 70 colorectal and 70 lung cancer samples from the experimental subset (Supplementary Table S1).

**Figure 1:**
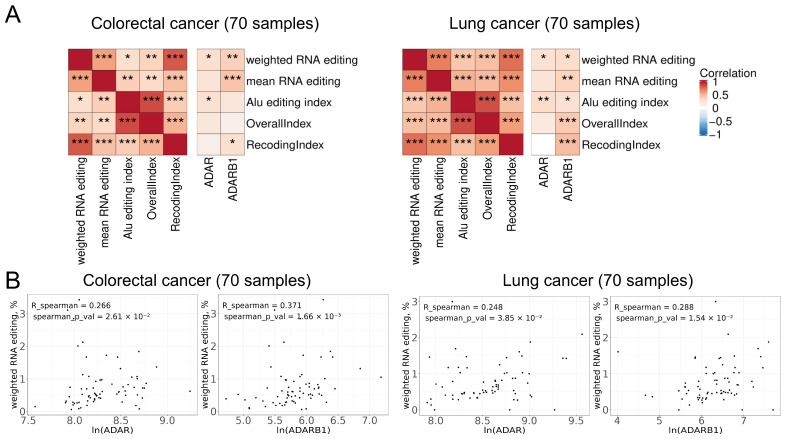
Comparison of RNA editing metrics. Correlation analysis of RNA editing metrics, including weighted RNA editing, mean RNA editing, overall editing level (OverallIndex), recoding editing index (RecodingIndex, REI), and Alu editing index (AEI). (**A**) Correlations among the metrics themselves (left panel) and with the expression of *ADAR* and *ADARB1* (right panel). *—*p* < 0.05, **—*p* < 0.01, ***—*p* < 0.001, Spearman’s rank correlation. (**B**) The relationship of the weighted RNA editing metric with *ADAR* and *ADARB1* expression in colorectal (left panels) and lung (right panels) cancers.

As a result of the correlation analysis of different RNA editing metrics, we observed positive correlations among all analyzed editing measures (Figure 1A). Weighted RNA editing, defined as the ratio of the total number of G reads to the total number of A + G reads across 24 editing sites, showed statistically significant positive correlations with the expression of both *ADAR1* (official gene ID *ADAR)* and *ADAR2* (official gene ID *ADARB1*) in colorectal and lung cancers (Figure 1A,B). The mean RNA editing metric exhibited a significant positive correlation with *ADARB1* expression in both cancer types, while its association with *ADAR* was positive but did not reach statistical significance. In addition, the Alu editing index, OverallIndex, and RecodingIndex showed either no correlation or statistically significant associations with only one of the *ADAR*/*ADARB1* genes (Figure 1A).

Analysis of the computational time required for each metric revealed that the weighted and mean RNA editing metrics exhibited substantially faster runtimes (~5 min per sample), whereas calculation of the AEI (RNAEditingIndexer, default settings) required on average 20–30 min per sample. In contrast, computation of the OverallIndex (REDItools2 + QEdit, default settings) required 120–140 min, and the RecodingIndex (REDItools2 + QEdit, default settings) required 70–80 min per sample (Supplementary Figure S1).

Thus, although mean RNA editing is calculated as the arithmetic mean of site-specific editing ratios, thereby assigning equal weight to each curated editing site and reducing expression-related variability, it showed no significant correlation with *ADAR* expression in both colorectal and lung cancer. In contrast, weighted RNA editing exhibited positive statistically significant correlations with *ADAR* and *ADARB1* expressions in both cancer types and demonstrated one of the highest computational efficiencies. In addition, using a set of 24 high-confidence sites also diminishes noise that could arise from genetic polymorphisms (the absence of genetic A/G polymorphisms in all 24 selected sites was confirmed using whole exome sequencing data of both experimental and TCGA datasets). For these reasons, weighted RNA editing was selected as the primary metric for all downstream functional analyses.

### 2.2. Overview of RNA Editing Patterns

**Figure 2:**
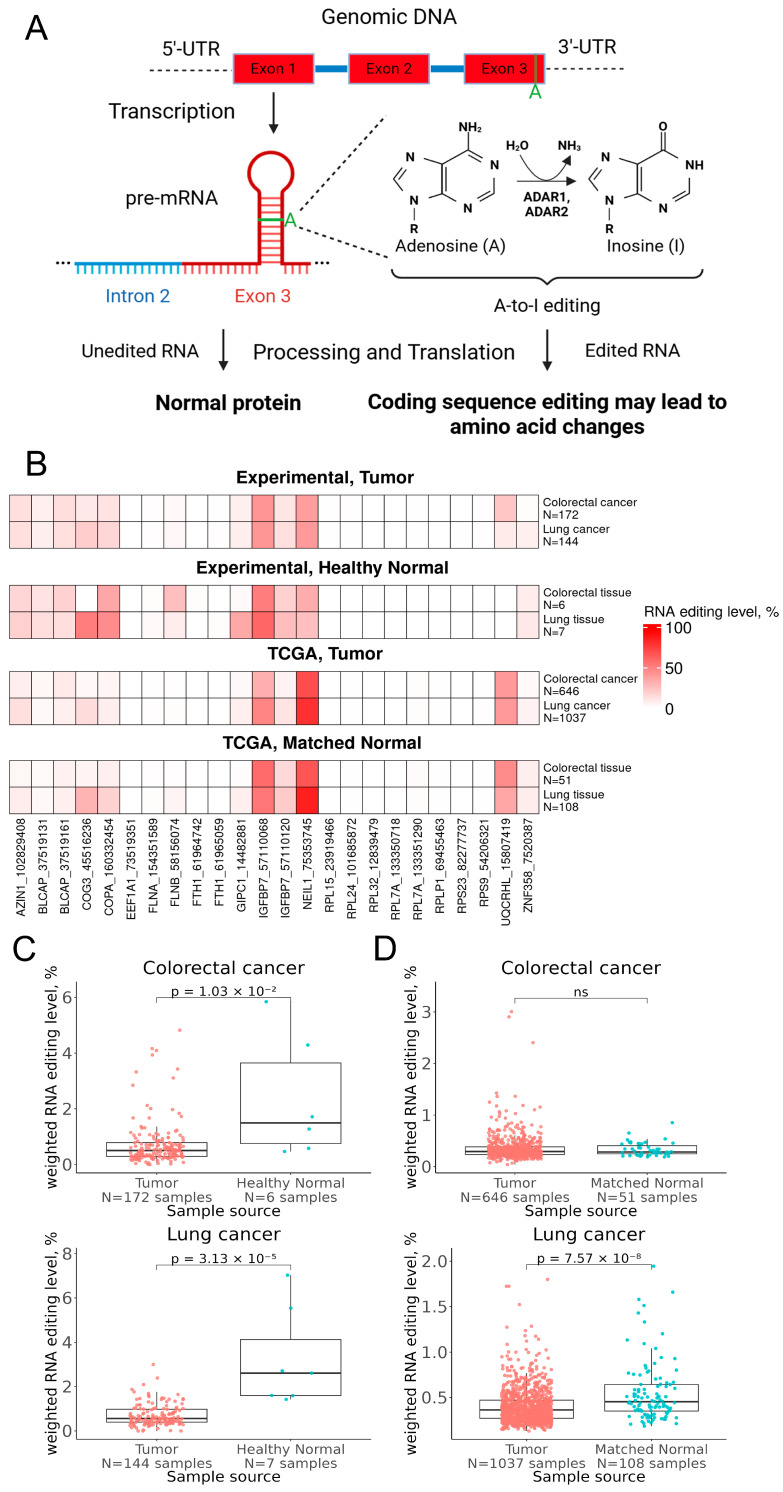
Overall analysis of RNA editing pattern. (**A**) Schematic representation of functional consequences of A-to-I(G) mRNA editing affecting amino acid coding sequences. (**B**) RNA editing level (G/(A + G)) by specific hotspot positions in experimental and TCGA tissue specimens. Gene IDs are shown with genomic positions of the RNA editing sites. The boxplot panels show overall RNA editing levels in cancer and normal tissues for experimental (**C**) and TCGA (**D**) samplings.

ADAR-mediated RNA editing can affect coding sequences, contributing to increased proteome diversity during oncogenesis (Figure 2A) [29]. Here we quantified RNA editing activity using a curated panel of 24 of the most frequently edited exonic loci in human genes, representing well-known substrates of ADAR enzymes. In the previous tests, these loci exhibited recurrent editing across the majority of investigated samples and, therefore, may serve as reliable markers of global ADAR activity. On the basis of editing levels at these sites, we constructed a composite RNA editing index, introduced in this study, to summarize RNA editing activity at the sample level.

Here we assessed ADAR-mediated editing for the groups of experimental and public TCGA RNA sequencing profiles of colorectal and lung cancers in comparison with the respective normal tissues. For TCGA, the controls were pathologically normal tissues adjacent to tumors; for the experimental samples, the controls were autopsy samples obtained from unrelated healthy donors killed in road accidents and profiled using the same equipment and protocols. A-to-G (T-to-C) editing events were deduced from RNA sequencing data and confirmed by whole exome sequencing data to be not related to possible cancer somatic mutations or specific germline variants.

Among the 24 analyzed editing sites, several loci consistently exhibited the highest mean editing levels across both tumor and normal samples (Figure 2B). In particular, editing events within the *NEIL1* (K242R) and *IGFBP7* (K95R) transcripts were among the most prominent in both cohorts.

In the experimental cohort, the mean editing levels in colorectal and lung cancer were 38.3% and 39.8% for *NEIL1*, and 41.9% and 41.1% for *IGFBP7*, respectively. In contrast, in healthy normal tissues, the mean editing levels were 33.3% and 25.8% for *NEIL1*, and 51.4% and 59.7% for *IGFBP7*. In tumor samples from TCGA, the corresponding mean editing levels in colorectal and lung cancer were 69.6% and 80.0% for *NEIL1*, and 31.9% and 48.2% for *IGFBP7*, respectively. In tumor-adjacent normal tissues, it reached 66.1% and 85.8% for *NEIL1*, and 58.0% and 54.1% for *IGFBP7*. Overall, for both colorectal and lung cancer, *NEIL1* (K242R) exhibited the lowest editing levels in healthy normal tissues. In contrast, *IGFBP7* (K95R) showed an opposite pattern, with maximal editing levels observed in healthy tissues and remaining high in tumor-adjacent normal tissues. These observations are consistent with previous studies, where these genes have been implicated in cancer-associated RNA editing [27,32,33]. However, for most positions, the mean level of RNA editing did not exceed 5% in both cancer and normal tissues (Figure 2B).

We observed that the overall level of ADAR-mediated RNA editing was statistically significantly higher in the healthy normal tissues compared to the tumor samples: ~2.9 and ~4.7-fold smaller for colorectal and lung cancers, respectively (Figure 2C,D). This pattern was evident only in experimental datasets. In TCGA data, statistically significant differences were observed for lung cancer: RNA editing levels in tumor-adjacent tissues were ~1.25-fold higher than in tumor tissues. This may reflect shared molecular features between tumors and tumor-adjacent tissues, in contrast to healthy normal tissues. Indeed, unlike healthy autopsy tissues, tumor-adjacent specimens share many common molecular features with cancer tissues, e.g., strong inflammation molecular phenotype [34]. Overall, these findings suggest that cancer transformation is associated with a global reduction in RNA editing activity for the analyzed sites, possibly reflecting the loss of normal regulatory control over ADAR enzymes or altered cellular environments within the tumor.

### 2.3. Correlation of RNA Editing with Biological Processes Reflecting DNA Integrity

**Figure 3:**
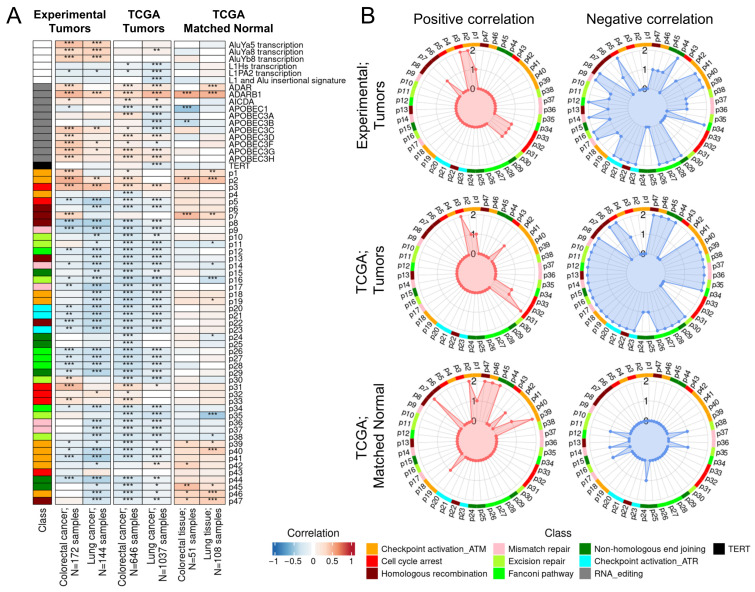
Correlation analysis of weighted RNA editing levels with DNA integrity parameters. (**A**) Correlation of RNA editing with biological processes reflecting DNA integrity in experimental and TCGA tumor and normal samples for colorectal and lung cancers. (**B**) Radar charts showing positive (left) and negative (right) correlations of ADAR-mediated RNA editing with DNA repair pathway activation features. p1–p47—DNA repair pathway IDs (full names given in Supplementary Table S2); *—*p* < 0.05, **—*p* < 0.01, ***—*p* < 0.001; Spearman’s rank correlation.

We then investigated the association of RNA editing with the processes related to DNA integrity (Figure 3). To this end we explored the correlation of the integral score of ADAR-mediated RNA editing with the following features: activation levels of DNA repair pathways, transcription of ADAR and APOBEC3 enzyme genes, expression of active families of human transposable elements (L1Hs, L1PA2, AluYa5, AluYa8, and AluYb8) and their insertional signature, and expression of the telomerase catalytic subunit gene *TERT*.

We detected positive correlations of ADAR-mediated RNA editing with the expression levels of *ADAR* and especially of *ADARB1* genes (Figure 3A), but no correlation or mixed correlation pattern with the expression of *APOBEC* family genes (Figure 3A). No clear-cut connection was found also for the *TERT* expression. In turn, the expression of L1 transposable elements, particularly L1PA2, showed a negative correlation with ADAR-mediated RNA editing in both experimental sampling and TCGA. However, no consistent pattern was detected for the L1 and Alu insertional signature. Similarly, no common trend was observed for the expression of Alu retrotransposons (Figure 3A). Taken together, these findings suggest a potential functional interconnection between RNA editing and the expression of *ADARB1* gene in both cancer and normal tissues under analysis, as well as the activities of L1 retrotransposons in cancer.

More consistent trends were seen for the activation levels of several DNA repair pathways (Figure 3). Most of the DNA repair pathways showed negative correlation, including five Fanconi anemia pathways (p12, 26–28, 34), three mismatch DNA repair pathways (p9, 14, 17), three ATM-mediated checkpoint activation pathways (p39, 40, 41), three ATR-mediated checkpoint activation pathways (p20, 21, 23), two non-homologous end joining pathways (p29, 44), two homologous recombination pathways (p8, 22), and one nucleotide excision pathway (p16). Interestingly, no negatively correlated pathways common to both cancer types were observed in matched normal samples.

In contrast, we detected positive correlations with ADAR-mediated RNA editing levels for a pathway involved in cell cycle arrest in response to DNA damage (*ATM Pathway G2/Mitosis Progression*, p3). In matched normal samples, positive correlations were observed for several pathways, including three ATM-mediated checkpoint activation pathways (p2, 39, 46), two homologous recombination pathways (p7, 47) and one non-homologous end joining pathway (p45).

**Figure 4:**
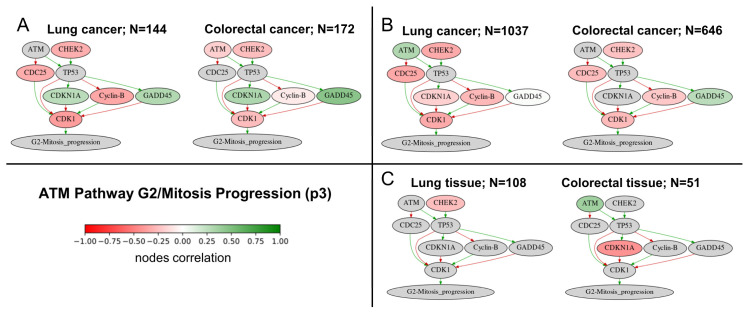
Schematic representation of the *ATM Pathway G2/Mitosis Progression* (p3) positively correlated with weighted RNA editing levels. Correlation of ADAR-mediated RNA editing levels with p3 components in experimental (**A**) and TCGA (**B**) cancer samples, and in TCGA tumor-adjacent normal tissues (**C**). Color intensity reflects statistically significant correlations, green for positive, red for negative, grey denotes the absence of statistically significant correlations. Green arrows represent activation, whereas red arrows indicate inhibition. For nodes containing more than one gene, statistically significant correlation values were averaged using Fisher z-transformation.

A more detailed analysis of the positively correlated pathway *ATM Pathway G2/Mitosis Progression* revealed that, uniquely in tumors, RNA editing levels were positively correlated with the expression of *GADD45* family genes inhibiting cell cycle progression, while were negatively correlated with genes promoting mitotic entry, such as *CDK1* and Cyclin B (*CCNB*) family genes (Figure 4). This finding suggests a link between ADAR-mediated editing and stress-responsive cell cycle regulation. Interestingly, no correlations with the expression of genes in this pathway that were common to both cancer types were observed in matched normal samples (Figure 4C).

We then evaluated the association between weighted RNA editing levels and specific indicators of genomic instability, including tumor mutational burden (TMB), copy number variation (CNV), loss of heterozygosity (LOH), number of fusion oncogenes, and microsatellite instability (MSI). MSI status was assessed exclusively in colorectal cancer samples, whereas the remaining genomic instability metrics (TMB, CNV, LOH, and number of fusion oncogenes) were analyzed in both colorectal and lung cancer cohorts. CNV, LOH, and fusion oncogene information was not available for the experimental dataset. For the literature-derived cohort, all genomic instability measures (TMB, MSI, CNV, LOH, and fusion oncogene profiles) were obtained directly from the TCGA project database.

Using a correlation coefficient threshold of 0.2 and a significance level of *p* < 0.05, we did not observe strong associations between RNA editing and genomic instability features in either the experimental or TCGA cohorts (Supplementary Figures S2 and S3).

In the experimental dataset, colorectal cancers with MSI showed elevated RNA editing levels compared with microsatellite stable (MSS) tumors, whereas no statistically significant differences were detected in the TCGA cohort (Supplementary Figure S4).

### 2.4. Genes Associated with RNA Editing

**Figure 5:**
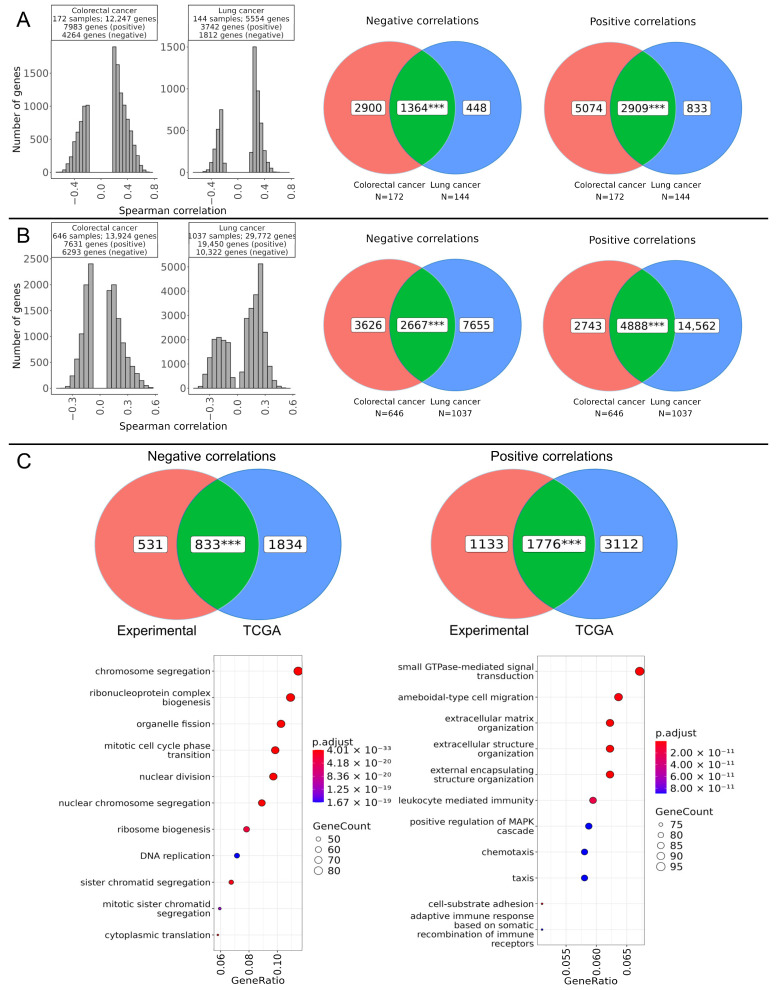
Genes correlated with weighted RNA editing levels in colorectal and lung cancer. Correlation and intersection analysis of ADAR-mediated RNA editing levels with RNA expression of 36,596 genes in experimental (**A**) and TCGA (**B**) colorectal and lung cancer samples. For the correlation analysis, Benjamini–Hochberg correction was applied (*p*-adjusted < 0.05). (**C**) Intersection of genes positively and negatively correlated with RNA editing that are common to both experimental and TCGA datasets. The statistical significance of the intersections was assessed using a permutation test. ***—*p* < 0.001.

We then performed a genome-wide correlation analysis between the level of ADAR-mediated RNA editing and the expression profiles of 36,596 genes. In both experimental and TCGA datasets, there were more positively than negatively correlated genes (Figure 5A,B). To identify genes that were consistently positively or negatively correlated across tumor types, genes from different cancer types and from experimental and TCGA datasets were intersected (Figure 5A–C). The number of common positively correlated genes was two-times greater than that of negatively correlated genes (1776 vs. 833, Figure 5C). Notably, *ADARB1*, one of the key RNA editing genes, was included in the list of double intersected positively correlated genes (Supplementary Table S3). Within the positively correlated intersections, we identified 121 transcription factor (TF) genes (Supplementary Table S4). The 12 TFs showing the strongest correlations with RNA editing levels are listed in Table 1 and represent diverse TF families, including ZBTB (3 genes), zf-C2H2 (2 genes), SAND (1 gene), Homeobox (1 gene), HMG (1 gene), T-box (1 gene), CSD (1 gene), bHLH (1 gene), and RHD (1 gene) families. This diversity of TFs suggests a complex regulatory landscape and highlights the multifaceted relationship between transcriptional regulation and RNA editing.

Furthermore, functional enrichment analysis of double intersected positively correlated gene sets revealed significant overrepresentation of pathways related to immune response, encompassing adaptive immunity (recombination of immune receptors, leukocyte mediated immunity). However, the strongest association was found with the categories related to remodeling of extracellular matrix and signal transduction (Figure 5C), which may reflect tissue reshaping during cancer progression.

In contrast, negatively correlated genes were enriched in processes associated with chromosome segregation, DNA replication, and mitotic cell cycle phase transition, in line with the previous findings of this study. Taken together, these results indicate that higher ADAR-mediated RNA editing activity tends to coincide with the activation of extracellular matrix remodeling and immune cell-related programs, whereas DNA replication and cell cycle phase transition are associated with reduced editing.

### 2.5. Molecular Pathways Associated with ADAR-Mediated RNA Editing

**Figure 6:**
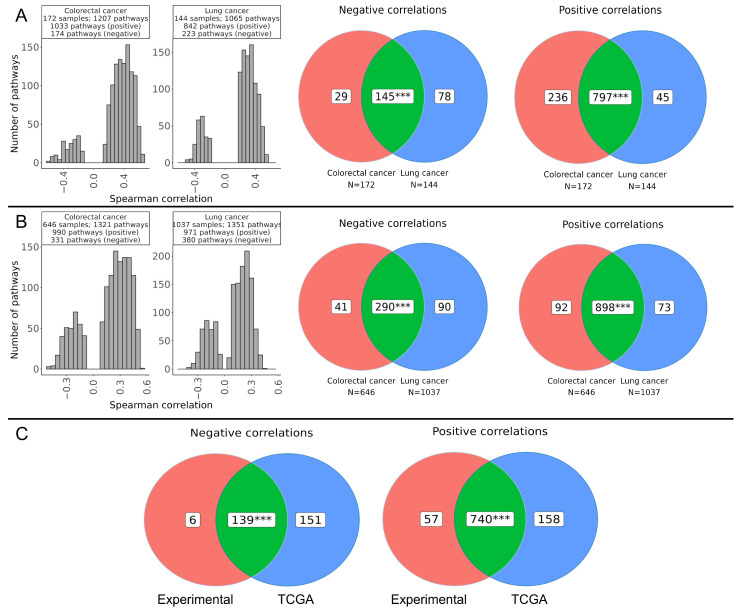
Molecular pathways correlated with weighted RNA editing levels in colorectal and lung cancer. Correlation and intersection analysis of ADAR-mediated RNA editing levels with pathway activation levels (PALs) of 1539 human molecular pathways in experimental (**A**) and TCGA (**B**) colorectal and lung cancer samples. For the correlation analysis, Benjamini–Hochberg correction was applied (*p*-adjusted < 0.05). (**C**) Intersection of molecular pathways positively and negatively correlated with RNA editing that are common to both experimental and TCGA datasets. The statistical significance of the intersections was assessed using a permutation test. ***—*p* < 0.001.

In addition to a previous gene-level assay, a similar correlation and intersection analysis was performed for the activation levels of 1539 human molecular pathways. Similar to gene-level analysis, more positively than negatively correlated pathways were identified for all the groups (Figure 6A,B).

We then identified double intersected correlating pathways common to both cancer types and to both experimental and TCGA datasets. In total, 740 positively correlated and 139 negatively correlated pathways were found (Figure 6C, Supplementary Table S5). Among them, the most strongly correlated hits are shown in Table 2. Notably, in the list of negatively correlated pathways (Supplementary Table S5) there was one of the key RNA editing pathways, *Reactome RIG-I MDA5 mediated induction of IFN-alpha/beta pathways Main Pathway*. This pathway involves the cytoplasmic RNA sensors RIG-I and MDA5, which recognize dsRNA and trigger a signaling cascade that leads to the production of type I interferons (IFN-α and IFN-β). ADAR edits A-to-I within dsRNA, modifying endogenous transcripts so that they are not recognized as foreign by RIG-I or MDA5. This editing effectively prevents aberrant activation of the interferon response by self-RNA. Otherwise, disruption of A-to-I editing can result in excessive RIG-I/MDA5 activation, increased IFN-α/β production, and the potential development of autoimmune or anti-tumor responses.

In addition, in good agreement with gene-level results, at the pathway level RNA editing appeared to be most strongly negatively correlated with several pathways involved in apoptosis (*GSK3 Signaling Pathway*, *Caspase Cascade Pathway*, and *Cellular Apoptosis Pathway*), immune sensing and inflammatory responses (*KEGG Tuberculosis Main Pathway*, *KEGG Herpes simplex infection Main Pathway*, *KEGG HTLV-I infection Main Pathway*, and *Glucocorticoid Receptor Signaling Pathway Inflammatory Cytokines*), and mRNA deadenylation (*Reactome Deadenylation of mRNA Main Pathway*) (Table 2, Supplementary Table S5). These observations suggest that generally, ADAR-mediated RNA editing in coding regions may be oppositely regulated with pathways controlling apoptosis, immune sensing, inflammatory responses, and mRNA degradation.

Positively correlated pathways showed overrepresentation of processes related to the extracellular matrix and cell signaling (Table 2, Supplementary Table S5). In particular, *ILK* (*Integrin Linked Kinase*) *Signaling*, *Reactome Molecules associated with elastic fibres Main Pathway*, *KEGG Focal adhesion Main Pathway*, and *KEGG ECM receptor interaction Main Pathway* related to extracellular matrix and respective intracellular regulation, in line with the gene-level analysis results (Figure 5C). Four other strongly positively correlated pathways deal with the regulation of proliferation and survival (*Ras Pathway*, *MAPK Signaling Pathway*, *ERK Signaling Pathway*, and *KEGG Ras signaling Main Pathway*). The positive correlation of RNA editing with these pathways may suggest that active A-to-I editing may be coordinated with cell growth signaling and adhesion to extracellular matrix. Thus, cells with high levels of ADAR-mediated RNA editing potentially exhibit increased plasticity and adaptability, which may be highly relevant to tumorigenesis.

## 3. Discussion

In this study, we propose a new metric for estimating RNA editing activity mediated by ADAR1 (encoded by the *ADAR* gene) and ADAR2 (encoded by the *ADARB1* gene) based on a targeted set of 24 hotspot editing positions in tumor and normal tissues. The existing strategies typically rely on calculating the mean editing level across all detectable sites, a process that is both time-consuming and resource-intensive. At the same time, numerous studies have shown that most RNA editing events occur within non-coding regions, where the presence of RNA modifications, such as methylation, can substantially increase sequencing error rates [4,14,35]. Moreover, elevated ADAR activity can lead to hyper-editing within Alu repeats, creating densely edited regions that further complicate comprehensive genome-wide quantification.

An established quantitative measure, the Alu editing index (AEI), is commonly used to assess global RNA editing activity and enables comparison of editing levels across samples [3,31]. The AEI quantifies A-to-I editing within Alu elements by calculating a weighted ratio of A-to-G mismatches to the total number of adenosines (A-to-G mismatches plus A-to-A matches) within predefined Alu-containing regions [31,35]. However, Alu elements are highly abundant [36] and divergent, which results in frequent low-confidence alignments in RNA-seq data [37] and can distort estimates of global editing levels. Similar to the OverallIndex and RecodingIndex, calculation of the AEI requires substantially more computational time than weighted and mean RNA editing metrics (Supplementary Figure S1). In addition, weighted RNA editing exhibited positive correlations with *ADAR* and *ADARB1* expression in both analyzed cancer types (Figure 1). Notably, the weighted RNA editing metric, along with mean RNA editing and RecodingIndex, showed stronger correlations with *ADARB1* expression, consistent with the established role of *ADARB1* as the primary contributor to RNA editing within coding sequences [30]. Because this metric is derived from a panel of frequently edited genes, it provides a more robust and reliable approximation of overall RNA editing activity and is additionally resilient to the confounding effects of genetic polymorphisms. Nevertheless, further studies are required to mitigate potential biases arising from uneven sequencing depth, as positions with higher read coverage depth can disproportionately influence the resulting metrics.

It is important to take into account that the use of a weighted editing score derived from 24 high-confidence positions, as well as any other type of aggregated scoring, while robust for cross-sample comparison, may obscure particular site-specific regulatory effects. Based on these positions, higher weighted RNA editing levels observed in healthy normal tissues, together with intermediate levels in tumor-adjacent tissues (Figure 2C,D), suggest that malignant transformation disrupts normal regulatory control of ADAR enzymes and/or reflects altered cellular environments within tumors. It should also be noted that, within the experimental cohort, a subset of control tissues was obtained from autopsies. Accordingly, potential postmortem effects should be considered, since postmortem interval and autolytic processes may influence RNA integrity and partially affect measured gene expression levels.

Our integrative analysis of RNA editing across experimental and TCGA colorectal and lung cancer datasets revealed that ADAR-mediated RNA editing is intricately associated with a diverse spectrum of genes and molecular pathways, reflecting its complex role in the cell. Among the positively correlated genes, we identified a group of 121 transcription factor (TF) genes, with the most strongly correlated and prevalent ones belonging to the ZBTB family (three genes: *HIC1*, *ZBTB47*, and *ZBTB46*) and the zf-C2H2 family (two genes: *GLIS2* and *KLF2*) (Table 1; Supplementary Table S4). Notably, the long non-coding RNA LINC01133, which is upregulated in non-small cell lung cancer tissues, has been reported to repress *KLF2* transcription through binding to EZH2 and LSD1 [38]. Given that non-coding RNAs are common targets of ADAR-mediated RNA editing [28], we hypothesize that elevated RNA editing levels may interfere with LINC01133-mediated repression, potentially restoring normal *KLF2* transcription. Interestingly, a recent study demonstrated that many C2H2 zinc-finger TFs possess RNA-binding activity [39]. This property may modulate the availability of double-stranded RNA regions, the substrates for ADAR enzymes, that influence the recruitment of ADAR to specific transcripts [39].

Additionally, gene ontology (GO) analysis of genes positively correlated with ADAR-mediated editing revealed an association between RNA editing and extracellular matrix (ECM)-related processes (Figure 5C). The ECM plays important structural and functional role in the tumor microenvironment. Abnormal changes in the ECM contribute to tumor invasion and metastasis, while alterations in cell signaling drive tumor growth and immune escape [40,41]. Although our analysis revealed positive correlations between RNA editing levels and well-known ECM genes, including collagens, ADAMTS, and MMPs (Supplementary Table S3), direct links between RNA editing and ECM-related genes have not yet been reported in the literature. This observation may point to a more complex regulatory relationship between RNA editing and ECM remodeling, potentially mediated through the editing of upstream regulators or non-coding RNAs. Notably, a gene from the developed panel, filamin A (*FLNA*), which plays a key role in cytoskeletal organization and cell-ECM interactions, also showed a positive correlation with weighted RNA editing levels (Supplementary Table S3). In vitro experiments demonstrated that the edited version of *FLNA* increases cellular stiffness and adhesion in mouse fibroblasts and human tumor cells [42]. Moreover, in a study using mouse models expressing either constitutively edited or uneditable *FLNA*, it was shown that RNA editing of *FLNA* in tumors may influence metastatic behavior by altering its interactions with the extracellular matrix [43]. However, further studies are required to elucidate both the direct and indirect effects of ADAR on extracellular matrix remodeling and cytoskeletal organization in tumors.

Positively correlated pathways are also predominantly linked to adaptive immunity and cellular signaling (Table 2, Supplementary Table S5). In particular, a study on B lymphopoiesis demonstrated that ADAR1 is essential for B cell maturation at the pro-B and pre-B stages through both MDA5-dependent and MDA5-independent mechanisms, where MDA5 functions as a cytosolic sensor of dsRNA and triggers immune signaling, leading to type I interferon responses [44]. Notably, the MDA5-dependent pathway regulates the early transition from pro-B to large pre-B cells, thereby supporting early B cell survival. MDA5-independent mechanisms control the subsequent progression from large to small pre-B cells by influencing the expression of pre-B cell receptors. Interestingly, RNA editing by ADAR enzymes can generate a distinct set of tumor-associated neoantigens, representing potential targets that could be exploited in immunotherapeutic strategies [45]. Moreover, a recent study demonstrated that peptides arising from ADAR-mediated RNA editing can be processed and presented by melanoma cells and subsequently recognized by tumor-infiltrating CD8^+^ T lymphocytes (TILs) [45]. In vivo observations indicated that lymphocytes specific to edited peptides accumulate within melanoma tumors and exert cytotoxic activity against cancer cells, implying that ADAR activity may have context-dependent consequences, simultaneously supporting tumor progression and promoting TIL-specific tumor cell elimination.

In thyroid cancer, it was shown that RAS induces *ADAR1* expression, an effect mediated primarily through the PI3K pathway and partially through MAPK signaling [46], which is consistent with our findings. In our study, RNA editing levels were also positively associated with activation of the *KEGG Focal adhesion Main Pathway*, consistent with findings in lung adenocarcinoma, where ADAR stabilizes the *FAK* transcript in an RNA editing-dependent manner, leading to activation of FAK signaling and promoting cancer cell migration and invasion [47]. Meanwhile, ADAR1 was shown to enhance A-to-I editing of miR-1251-5p in lung adenocarcinoma [48]. The edited miR-1251-5p exhibited a significantly stronger suppressive effect on tumor growth and metastatic potential compared with its unedited form.

In our study, RNA editing levels in tumors were positively correlated with the *ATM Pathway G2/Mitosis Progression*, whereas negative correlations were observed with ATM- and ATR-mediated checkpoint activation pathways, suggesting a complex role for RNA editing in cell cycle arrest in cancer (Figure 3). A study performed in HEK293 cells demonstrated that ADAR1, through the upregulation of *CDK2*, can decrease the proportion of cells in the G0/G1 phase, increased the percentage of cells in S phase, and did not alter the fraction of cells in G2/M [49]. In addition, inhibition of *ADAR1* expression in the A549 and H1299 cell lines resulted in an increased proportion of cells in the G0/G1 phase, highlighting the role of ADAR1 in cell cycle regulation [50]. It is important to consider the impact of site-specific RNA editing in the 3′UTRs of genes, as demonstrated in a study of breast cancer cell lines, where an RNA-edited site in the 3′UTR of *ATM* increased its expression [51].

On the other hand, one of the pathways most strongly inversely associated with RNA editing levels is the *Reactome RIG-I MDA5 mediated induction of IFN-alpha/beta pathways Main Pathway* (Table 2, Supplementary Table S5), which centers on the cytosolic RNA sensors RIG-I and MDA5, which detect dsRNA and initiate signaling events leading to type I interferon (IFN-α/β) production. A-to-I editing by ADAR enzymes alters endogenous dsRNA structures, thereby reducing their recognition by RIG-I and MDA5 and preventing inappropriate activation of interferon signaling by self-derived RNA. When this editing process is impaired, endogenous dsRNA can aberrantly activate RIG-I/MDA5, resulting in elevated IFN-α/β responses and potentially fostering autoimmune or anti-tumor immune activation [17,52,53,54,55].

Negatively correlated pathways are also associated with DNA repair, mRNA deadenylation, and apoptosis (Table 2, Supplementary Table S5). The inverse relationship between RNA editing and these genome maintenance pathways may reflect a functional trade-off in tumor cells, where enhanced post-transcriptional modification coincides with a relative reduction in canonical DNA repair. Multiple studies have demonstrated that ADAR-mediated RNA editing modulates mRNAs of proteins associated with DNA repair and cell cycle regulation, revealing a potential new role for ADAR in tumorigenesis and cancer progression. In the study [56], ADAR1 may indirectly inhibit *CDKN1A* expression via its effect on microRNA miR-26a, thereby accelerating cell cycle progression and cell proliferation, and ADAR1-mediated modification of the *MDM2* 3′UTR disrupts miRNA binding and downregulates *TP53* transcription. In both non-small cell lung cancer and multiple myeloma, upregulated *ADAR1* expression has been shown to elevate A-to-I editing at the K242R site within the *NEIL1* transcript, an event that converts lysine to arginine at position 242, which in turn enhances tumor cell proliferation and contributes to therapeutic resistance [57,58]. The NEIL1 protein with K242R editing shows a diminished capacity to repair oxidative DNA lesions, resulting in the accumulation of double-strand breaks. Consequently, modification at this site may increase tumor susceptibility to therapies that generate both single- and double-strand DNA damage [57]. The negative association between RNA editing levels and DNA repair and apoptotic pathways suggests that increased RNA editing may contribute to impaired cellular responses to genomic damage rather than directly driving genome instability. The modest enrichment of RNA editing observed in colorectal cancers with microsatellite instability (MSI), together with the weak or absent correlations with tumor mutational burden (TMB), copy number variation (CNV), loss of heterozygosity (LOH), and number of fusion oncogenes, is consistent with this interpretation, as MSI primarily reflects defects in the DNA mismatch repair (MMR) system (Supplementary Figures S2–S4). However, further studies of RNA editing across additional cancer types are needed to more precisely define its role in DNA repair and apoptosis during tumor progression [59,60].

In addition, another study reported that A-to-I RNA editing can influence and alter the mechanisms of double-strand DNA breaks repair, involving numerous RNA-based factors, such as DNA:RNA hybrids often observed near broken sites on DNA [61]. These hybrids are targeted by RNA editing enzymes, especially ADAR2 [62]. The mismatches generated by RNA editing reduce the stability of RNA-DNA base pairing, facilitating unwinding by the helicase activity of SETX and promoting DNA resection. However, further studies are needed to clarify the relationship between RNA editing and DNA repair mechanisms as well as cell cycle regulation.

**Figure 7:**
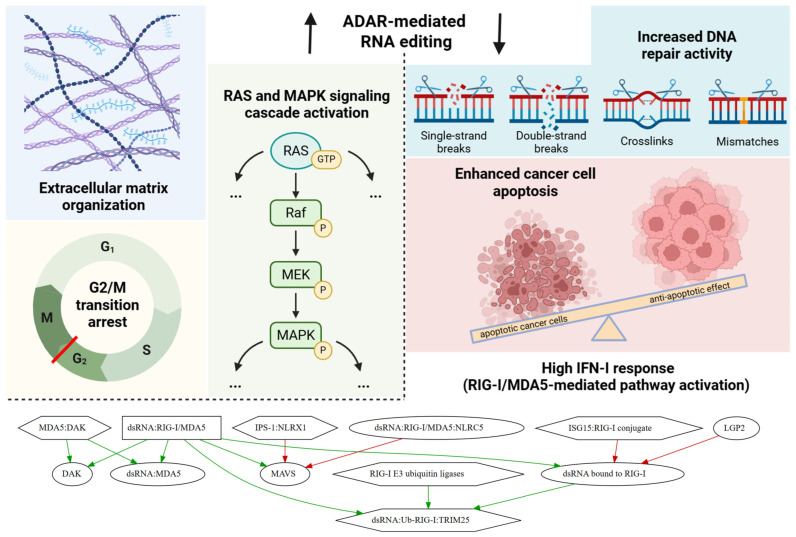
Main associations of RNA editing levels in cancer cells. In this study, elevated levels of RNA editing were associated with extracellular matrix remodeling, RAS and MAPK signaling cascade activation, and cell cycle arrest via the *ATM Pathway G2/Mitosis Progression* checkpoint (p3). In contrast, reduced RNA editing activity corresponded to enhanced DNA repair, increased cancer cell apoptosis, and a heightened type I interferon (IFN-I) response. Green arrows represent activation, whereas red arrows indicate inhibition.

Together, these observations underscore the dual role of RNA editing in cancer. On one hand, it appears to support tumor-promoting processes, such as ECM remodeling, RAS and MAPK signaling pathway activation, reduced apoptosis, and diminished IFN-I response (Figure 7). On the other hand, its inverse relationship with genome maintenance pathways suggests that RNA editing may contribute to tumor heterogeneity and adaptability by modulating the balance between RNA editing level, DNA repair, and cell cycle progression. These findings highlight the potential of RNA editing as both a biomarker and a regulatory node in the complex network of molecular processes driving tumorigenesis. Despite the limited number of single-cell studies on RNA editing [63,64], the application of such technologies holds great promise for resolving cell type-specific RNA editing patterns. In particular, profiling RNA editing in single cells from lung adenocarcinoma biopsy samples revealed that the increased editing observed in bulk lung tumors was specific to cancer cells [64]. Moreover, integrating the weighted RNA editing metric with mutational, methylation, and chromatin accessibility profiles could enable more precise identification of causal relationships between RNA editing and molecular processes in tumors.

Noteworthy, the important factor involved in the posttranscriptional regulation of transposable elements may be influenced by RNA editing enzymes. Despite the prevalence of Alu elements as targets for ADAR enzymes, this does not result in a substantial alteration of their RNA concentration [65]. Nevertheless, it has been previously demonstrated that for L1 elements *ADAR* and *ADARB1* function as negative regulators [66,67]. This observation aligns with our findings, which revealed a negative correlation between ADAR-mediated RNA editing levels and L1 features, particularly L1PA2 expression, in both experimental and TCGA datasets (Figure 3). Previous studies [66,68] showed that the catalytic ADAR activity is not required for L1 suppression, thus supporting potential alternative scenarios for the correlations.

## 4. Materials and Methods

### 4.1. Experimental Biosamples

The experimental biosamples included in this study were obtained from Oncobox clinical trials (Clinicaltrials.gov ID NCT03724097, NCT03521245) or submitted for Oncobox cancer molecular testing (www.oncobox.com) [69]. For each biospecimen, written informed consent for participation in this study was obtained from the patient or their legal representative. The consent process and study design were guided and approved by the local ethics committee of the Vitamed Clinic (Moscow, Russia).

The study comprised a total of 316 experimental solid cancer samples (Table 3). All experimental biosamples were human formalin-fixed paraffin-embedded (FFPE) tissue blocks, including 172 colorectal and 144 lung cancer specimens. The lung cancer cohort consisted of the following subtypes: lung adenocarcinoma (73 samples), lung squamous cell carcinoma (52 samples), and other lung cancer types, including small cell lung cancer, neuroendocrine carcinoma, and unclassified cases (19 samples). All tissue specimens were evaluated by a pathologist to confirm diagnosis and to assess content of tumor cells. Specific tumor regions exhibiting a minimum of 50% tumor cells were dissected for further extraction of DNA and RNA. For each tissue type, the corresponding healthy tissue biosamples from the ANTE database of healthy human tissue RNA-seq profiles [70] were used. Both experimental healthy control and tumor samples were profiled using the same gene library preparation and RNA sequencing protocol [70], yielding approximately 30 million raw reads per sample. Whole exome sequencing (WES) and variant calling of experimental samples were performed according to our previous protocol [71].

Pre-selected collection of TCGA colorectal and lung cancer samples with RNA sequencing and WES data and the corresponding tumor-matched adjacent pathologically normal tissues was taken from [34]. The colorectal cancer cohort included colon adenocarcinoma (COAD, 479 samples) and rectum adenocarcinoma (READ, 167 samples), whereas the lung cancer cohort consisted of lung adenocarcinoma (LUAD, 535 samples) and lung squamous cell carcinoma (LUSC, 502 samples).

For the comparison of the proposed and established RNA editing metrics, we analyzed 70 colorectal and 70 lung cancer samples from the experimental cohort (Supplementary Table S1).

### 4.2. Analysis of RNA Expression of Human Genes and Transposable Elements

RNA sequencing reads of experimental samples were aligned to the genome (Human genome assembly GRCh38) using STAR v2.6.1d software. Ensembl gene IDs were converted to HUGO Gene Nomenclature Committee (HGNC) gene symbols using the Complete HGNC dataset (database version from 13 July 2017). Totally, expression levels were established for 36,596 annotated genes with the corresponding HGNC identifiers. Expression of transposable elements was calculated using TEtranscripts v2.2.1 [72] with default settings. DESeq2 v1.44.0 was used to normalize gene expression profiles prior to further comparisons.

The transcriptional signature score of L1Hs, L1PA2, *TERT* and *APOBEC3B* expression levels (L1 and Alu insertional signature) was calculated as the sum of the normalized and log-transformed expression values according to [73].

### 4.3. Activation Levels of Intracellular Molecular Pathways

The pathway activation levels (PALs) of human molecular pathways were calculated using RNA sequencing data using Oncobox algorithm and OncoboxPD pathway database [74]. Pathways with more than 10 participating gene products were selected for further analysis (1539 pathways, including 47 DNA repair pathways). For PAL calculations, the expression level of each gene in a given sample was normalized by the geometric mean expression of this gene across all biosamples of the corresponding tissue type (tumor or normal) within the same dataset (experimental or TCGA) included in the analysis. PAL values were calculated as follows Equation (1):

(1) $${PAL}_{p}=100\times \sum_{n}{(ARR}_{n,p}\times lg({CNR}_{n}))/\sum_{n}{|ARR}_{n,p}|,$$

where PAL*_p_* is PAL for a pathway *p*; CNR*_n_* is the case-to-normal ratio for a gene *n*; ARR (activator/repressor role) is a Boolean value that depends on the function of this gene product in the pathway *p* [75]. ARR*_np_* is a Boolean value defined as follows: −1 when the product of the gene *n* inhibits the pathway *p*; 1 when the product of *n* activates *p*; 0 when the product of *n* has an ambiguous role in *p*. The CNR*_n_* value is calculated as the ratio of a quantitative metric level for the gene *n* in a biosample under study to an average level for *n* in the corresponding group (tumor or normal).

### 4.4. Assessment of RNA Editing Level

The extent of adenosine-to-inosine (A-to-I(G)) conversion by adenosine deaminase acting on RNA (ADAR) was quantitatively assessed using a set of top-20 representative genes edited in at least 80% of samples according to the REDIportal database [76]. The study includes 24 exonic sites in the *AZIN1*, *BLCAP*, *COG3*, *COPA*, *EEF1A1*, *FLNA*, *FLNB*, *FTH1*, *GIPC1*, *IGFBP7*, *NEIL1*, *RPL15*, *RPL24*, *RPL32*, *RPL7A*, *RPLP1*, *RPS23*, *RPS9*, *UQCRHL* and *ZNF358* genes [77], positions shown in Table 4. Variant allele frequencies were estimated using bam-readcount v1.0.1 [78] to calculate the average editing rate per site.

We evaluated several widely used metrics for estimating global RNA editing levels, including the overall editing level (OverallIndex) [79], the recoding editing index (RecodingIndex, REI) [80], and the Alu editing index (AEI) [31]. These metrics were calculated from STAR-derived BAM files with uniquely mapped high-quality reads (MAPQ ≥ 20).

The OverallIndex is defined as the ratio of sequencing reads containing G at known A-to-I editing positions to the total number of reads covering these positions, without imposing stringent sequencing coverage thresholds [79]. This metric was computed using 15,612,384 RNA editing sites annotated in REDIportal, with per-site editing levels derived from REDItools2 (https://github.com/BioinfoUNIBA/REDItools2, accessed on 26 December 2025) output tables and further processed using QEdit software (https://github.com/BioinfoUNIBA/QEdit, accessed on 26 December 2025) [77,79,81].

The RecodingIndex represents the overall editing level calculated specifically at recoding sites located within protein-coding genes [80]. Similar to the OverallIndex, the RecodingIndex was computed from REDItools2 output tables and processed using QEdit software, based on a curated list of 20,885 recoding sites retrieved from REDIportal [77,79,81].

The AEI quantifies RNA editing within Alu repetitive elements and is calculated as the ratio of edited reads exhibiting A-to-G mismatches to the total coverage of adenosine bases within Alu regions using RNAEditingIndexer v1.2 [31]. Formally, AEI corresponds to the weighted average of editing levels across all adenosines in Alu elements, where the weights are determined by the sequencing coverage at each position (i.e., the sum of A-to-G mismatches and A-to-A matches).

Despite differences in scope and implementation, all three metrics are conceptually based on estimating the proportion of edited guanosines relative to the total number of adenosines and guanosines (G/(A + G)) across predefined sets of editing sites. Thus, in line with these established metrics, we developed a coverage-weighted RNA editing score for a selected set of 24 edited sites. For each site, the mutation rate was calculated using bam-readcount with parameters -q 20 -b 20. This metric was defined as a weighted G/(A + G) ratio, where individual site-specific editing levels were weighted by their corresponding sequencing coverage Equation (2):

(2) $$weighted\;RNA\;editing\;level=\frac{\sum_{i=1}^{n}A>G_{cov,i}}{\sum_{i=1}^{n}\left(A>G_{cov,i}+A>A_{cov,i}\right)}\times 100,$$

where *n* is the number of analyzed sites; *i* denotes a specific site; *A* > *G_cov,i_* represents the number of reads supporting the A > G substitution (T > C on the reverse strand); *A* > *A_cov,i_* denotes the number of reads supporting the reference allele A (T on the reverse strand).

For each editing position, whole exome sequencing data of the respective biosample were manually inspected to exclude the possibility of A/G or T/C DNA mutation or genetic polymorphism in the corresponding site.

As an alternative RNA editing metric, the editing rate was also defined as the number of G reads at an A reference site (or C reads at a U(T) reference site on an opposite strand), divided by the total number of A + G (T + C) reads mapped to the site. For each site, the mutation rate was calculated using bam-readcount with parameters -q 20 -b 20, considering only sites with a coverage of more than five A + G (T + C) reads, as follows Equation (3):

(3) $$mean\;RNA\;editing\;level=\frac{1}{n}\sum_{i=1}^{n}\frac{A>G_{cov,i}}{A>G_{cov,i}+A>A_{cov,i}}\times 100,$$

where *n* is the number of analyzed sites that passed the five-nucleotide filter; *i* denotes a specific site; *A* > *G_cov,i_* represents the number of reads supporting the A > G substitution (T > C on the reverse strand); *A* > *A_cov,i_* denotes the number of reads supporting the reference allele A (T on the reverse strand).

### 4.5. Microsatellite Instability

Microsatellite instability (MSI) was inferred from transcriptomic profiles following previously published approaches [82,83]. Two expression-based composite metrics were generated. The first metric reflected functional impairment of the DNA mismatch repair (MMR) system and was calculated as the negative sum of log-transformed expression values of the core MMR genes (*MLH1*, *MSH2*, *MSH6*, and *PMS2*). The second metric captured a hypermutator transcriptional signature and was obtained as a weighted linear combination of log-transformed expression values of a predefined gene signature associated with hypermutator phenotypes (*EPM2AIP1*, *TTC30A*, *SMAP1*, *RNLS*, *WNT11*, *SFXN1*, *SREBF1*, *TYMS*, *EIF5AL1*, and *WDR76*), with gene-specific coefficients adopted from [83]. MSI status was subsequently assigned by combining both metrics within a linear classification framework, whereby samples were categorized as MSI-positive or MSI-negative (microsatellite stable, MSS).

### 4.6. WES Data Processing

WES data were analyzed using a standardized workflow adapted from previously reported protocols [84]. Sequencing reads were mapped to the human reference genome (GRCh38) with BWA mem v0.7.17 [85]. BAM files were indexed using Samtools v1.3.1 [86], followed by duplicate read identification with the MarkDuplicates module from the Picard toolkit (http://broadinstitute.github.io/picard) (accessed on 15 September 2025). Base quality score recalibration was performed using the BaseRecalibrator and ApplyBQSR tools implemented in GATK v4.beta.1. Mutation calling in tumor samples was carried out using Mutect2 [87], with known variant resources, including the 1000G gold standard indel set [88] and the dbSNP database [89]. GATK4 FilterMutectCalls and ANNOVAR v2017 tools were applied for variant filtering and annotation.

Tumor mutation burden (TMB) was calculated for both experimental and TCGA datasets as the total number of somatic coding variants, including single-nucleotide substitutions and insertions/deletions, normalized to the size of the coding exome (mutations per megabase).

### 4.7. Statistical Analysis and Visualization

Spearman correlation coefficients with *p*-values were calculated using the R “stats” package (version 4.4.0) and visualized with the R “pheatmap” package (version 1.0.12). Other plots were generated with the R “ggplot2” package (version 3.5.1).

Gene Ontology (GO) enrichment analysis was performed with the R packages “clusterProfiler” (version 4.2.1) and “org.Hs.eg.db” (version 3.8.2). Venn diagram plotting was performed using the R packages “ggVennDiagram” (version 1.5.2) and “RVenn” (version 1.1.0). Molecular pathways were visualized using the OncoboxPD toolkit [74].

Comparisons of RNA editing levels between different groups were performed using the non-parametric Mann–Whitney U test in R v4.4.0 (Benjamini–Hochberg adjusted *p*-value threshold 0.05).

## 5. Conclusions

In summary, the quantification strategy based on weighted RNA editing levels across 24 hotspot ADAR target sites in coding regions provides a robust and scalable framework for assessing RNA editing activity by ADAR1 and ADAR2 in large transcriptomic datasets. This approach effectively captures global editing trends while minimizing the noise introduced by site-specific variability. Our study demonstrates that ADAR-mediated RNA editing is tightly intertwined with several fundamental molecular processes governing cell physiology. The strong positive correlations between global editing levels and pathways related to extracellular matrix organization, RAS-MAPK cascade signaling and cell cycle arrest at G2/M phase, and negative correlations with IFN-I response, DNA repair, mRNA deadenylation, expression of transposable elements and apoptosis highlight the integrative nature of ADAR activity within the cellular network. In the future this analysis can be broaden to assess more cancer types and integrate single-cell transcriptomic and epitranscriptomic datasets to resolve cell type-specific patterns of RNA editing. Combining RNA editing metrics with mutational, methylation, and chromatin accessibility profiles could provide a more comprehensive understanding of the interconnection of multiple layers of genome regulation in cancer cells.

## Figures and Tables

**Table 1:** Double intersected TF genes most strongly correlated with ADAR-mediated RNA editing level.

Gene ID	TF Family	Mean Correlation Value (Experimental)	Mean Correlation Value (TCGA)
*CSDC2*	CSD	0.43	0.43
*GLIS2*	zf-C2H2	0.57	0.45
*HIC1*	ZBTB	0.51	0.51
*HLX*	Homeobox	0.44	0.46
*KLF2*	zf-C2H2	0.53	0.39
*LYL1*	bHLH	0.42	0.44
*NFATC1*	RHD	0.55	0.29
*SAMD11*	SAND	0.49	0.43
*SOX18*	HMG	0.49	0.37
*TBX2*	T-box	0.47	0.39
*ZBTB46*	ZBTB	0.47	0.39
*ZBTB47*	ZBTB	0.54	0.36

**Table 2:** Molecular pathways most strongly correlated with RNA editing.

Top-12 Positively Correlated Pathways ^1^	Mean Correlation ^2^	Top-12 Negatively Correlated Pathways ^1^	Mean Correlation ^2^
*ILK Signaling Pathway*	0.529	*GSK3 Signaling Pathway*	−0.496
*MAPK Signaling Pathway*	0.528	*Caspase Cascade Pathway*	−0.491
*KEGG Focal adhesion Main Pathway*	0.526	*Cellular Apoptosis Pathway*	−0.474
*Ras Pathway*	0.509	*KEGG Tuberculosis Main Pathway*	−0.441
*Reactome Molecules associated with elastic fibres Main Pathway*	0.504	*KEGG Herpes simplex infection Main Pathway*	−0.429
*KEGG Rap1 signaling Main Pathway*	0.502	*KEGG HTLV-I infection Main Pathway*	−0.425
*Estrogen Pathway*	0.500	*cAMP Pathway Endothelial Cell Regulation*	−0.409
*KEGG ECM receptor interaction Main Pathway*	0.500	*KEGG Oocyte meiosis Main Pathway*	−0.406
*KEGG Ras signaling Main Pathway*	0.499	*Reactome Deadenylation of mRNA Main Pathway*	−0.406
*Reactome O-glycosylation of TSR domain containing proteins Main Pathway*	0.498	*KEGG Hippo signaling Main Pathway*	−0.397
*GPCR Pathway*	0.497	*Reactome RIG-I MDA5 mediated induction of IFN-alpha/beta pathways Main Pathway*	−0.390
*ERK Signaling Pathway*	0.496	*Glucocorticoid Receptor Signaling Pathway Inflammatory Cytokines*	−0.377

^1^ Sorted by absolute value of mean correlation. ^2^ Mean correlation calculated for experimental and TCGA samplings.

**Table 3:** Statistics of experimental and TCGA sequencing profiles.

Experimental Biosamples, Number of RNA Sequencing Profiles			
Tissue		Cancer Samples	Healthy Normal Samples
Colorectal cancer		172	6
Lung cancer	Adenocarcinoma	73	7
	Squamous cell carcinoma	52	
	Other types	19	
Total		316	13
**TCGA Biosamples, Number of RNA Sequencing Profiles**			
**Tissue**		**Cancer Samples**	**Adjacent (Tumor-Matched) Normal Samples**
Colorectal cancer	COAD	479	41
	READ	167	10
Lung cancer	LUAD	535	59
	LUSC	502	49
Total		1683	159
**Number of Whole Exome Sequencing Profiles**			
**Tissue**		**Experimental Tumor Samples**	**TCGA Tumor Samples**
Colorectal cancer		71	537
Lung cancer		81	1006
Total		152	1543

**Table 4:** Hotspot ADAR-mediated RNA editing sites investigated in this study.

Gene ID	Chromosome	Position (hg38)	Substitution Type	Gene ID	Chromosome	Position (hg38)	Substitution Type
*AZIN1*	chr8	102,829,408	nonsynonymous (S367G)	*IGFBP7*	chr4	57,110,120	nonsynonymous (R78G)
*BLCAP*	chr20	37,519,131	nonsynonymous (K15R)	*NEIL1*	chr15	75,353,745	nonsynonymous (K242R)
*BLCAP*	chr20	37,519,161	nonsynonymous (Q5R)	*RPL15*	chr3	23,919,466	nonsynonymous (R194G)
*COG3*	chr13	45,516,236	nonsynonymous (I635V)	*RPL24*	chr3	101,685,872	synonymous (P46P)
*COPA*	chr1	160,332,454	nonsynonymous (I164V)	*RPL32*	chr3	12,839,479	nonsynonymous (K50E)
*EEF1A1*	chr6	73,519,351	nonsynonymous (T104A)	*RPL7A*	chr9	133,350,718	nonsynonymous (Q206R)
*FLNA*	chrX	154,351,589	nonsynonymous (S2339G)	*RPL7A*	chr9	133,351,290	nonsynonymous (L242P)
*FLNB*	chr3	58,156,074	nonsynonymous (Q2296R)	*RPLP1*	chr15	69,455,463	nonsynonymous (S101P)
*FTH1*	chr11	61,964,742	synonymous (S179S)	*RPS23*	chr5	82,277,737	synonymous (P40P)
*FTH1*	chr11	61,965,059	synonymous (L105L)	*RPS9*	chr19	54,206,321	nonsynonymous (E89G)
*GIPC1*	chr19	14,482,881	synonymous (P32P)	*UQCRHL*	chr1	15,807,419	synonymous (A77A)
*IGFBP7*	chr4	57,110,068	nonsynonymous (K95R)	*ZNF358*	chr19	7,520,387	nonsynonymous (K382R)

## Data Availability

The original contributions presented in this study are included in the article/Supplementary Materials. Further inquiries can be directed to the corresponding author.

## Supplementary Materials

The following supporting information can be downloaded at: https://www.mdpi.com/article/10.3390/ijms27062625/s1.

## Abbreviations

The following abbreviations are used in this manuscript:

AEI	Alu editing index
CNV	Copy number variation
dsRNA	Double-stranded RNA
ECM	Extracellular matrix
FFPE	Formalin-fixed paraffin-embedded block
GO	Gene ontology
LOH	Loss of heterozygosity
MSI	Microsatellite instability
PAL	Pathway activation level
TF	Transcription factor
TILs	Tumor-infiltrating lymphocytes
TMB	Tumor mutation burden
UTR	Untranslated region
WES	Whole exome sequencing
