# Supplementary material for: Molecular Context of ADAR-Mediated Editing of Coding RNA in Colorectal and Lung Cancers
Source: Int J Mol Sci. 2026 Mar 13;27(6):2625. doi: 10.3390/ijms27062625 (PMC13027024; doi:10.3390/ijms27062625)
Supplement: Supplementary file 1 [file ijms-27-02625-s001.zip › Supplementary_Figures.pdf]

Article "Molecular Context of ADAR-Mediated Editing of Coding RNA in Colorectal and Lung Cancers"

Supplementary Figures

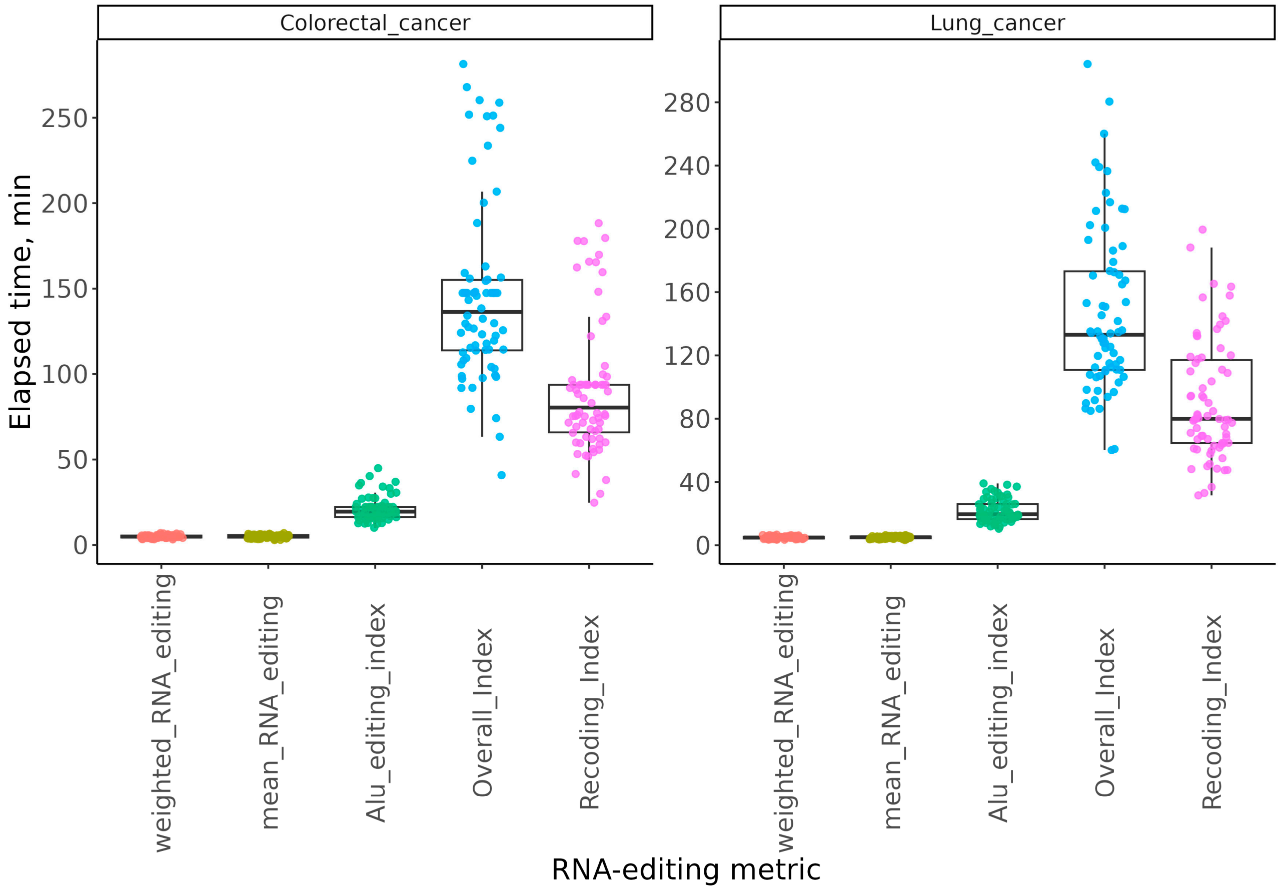

Figure S1. Analysis of the computational time required for the calculation of RNA editing metrics in 70 colorectal cancer samples (left panel) and 70 lung cancer samples (right panel). Elapsed time is expressed in minutes.

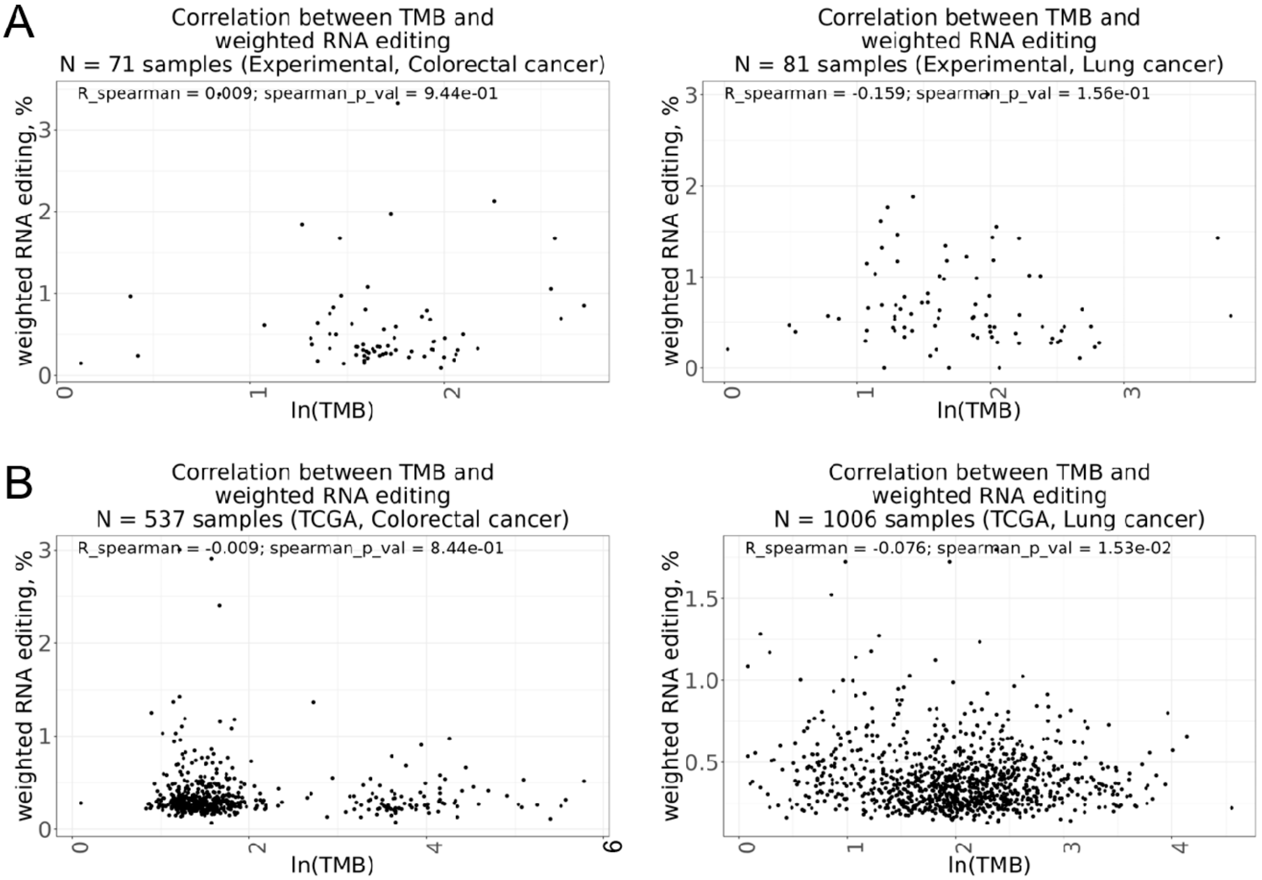

Figure S2. Spearman's rank correlation between tumor mutation burden (TMB) and the weighted RNA editing level in experimental (A) and TCGA (B) cancer samples. Colorectal cancer samples are shown in the left panels, and lung cancer samples in the right panels.

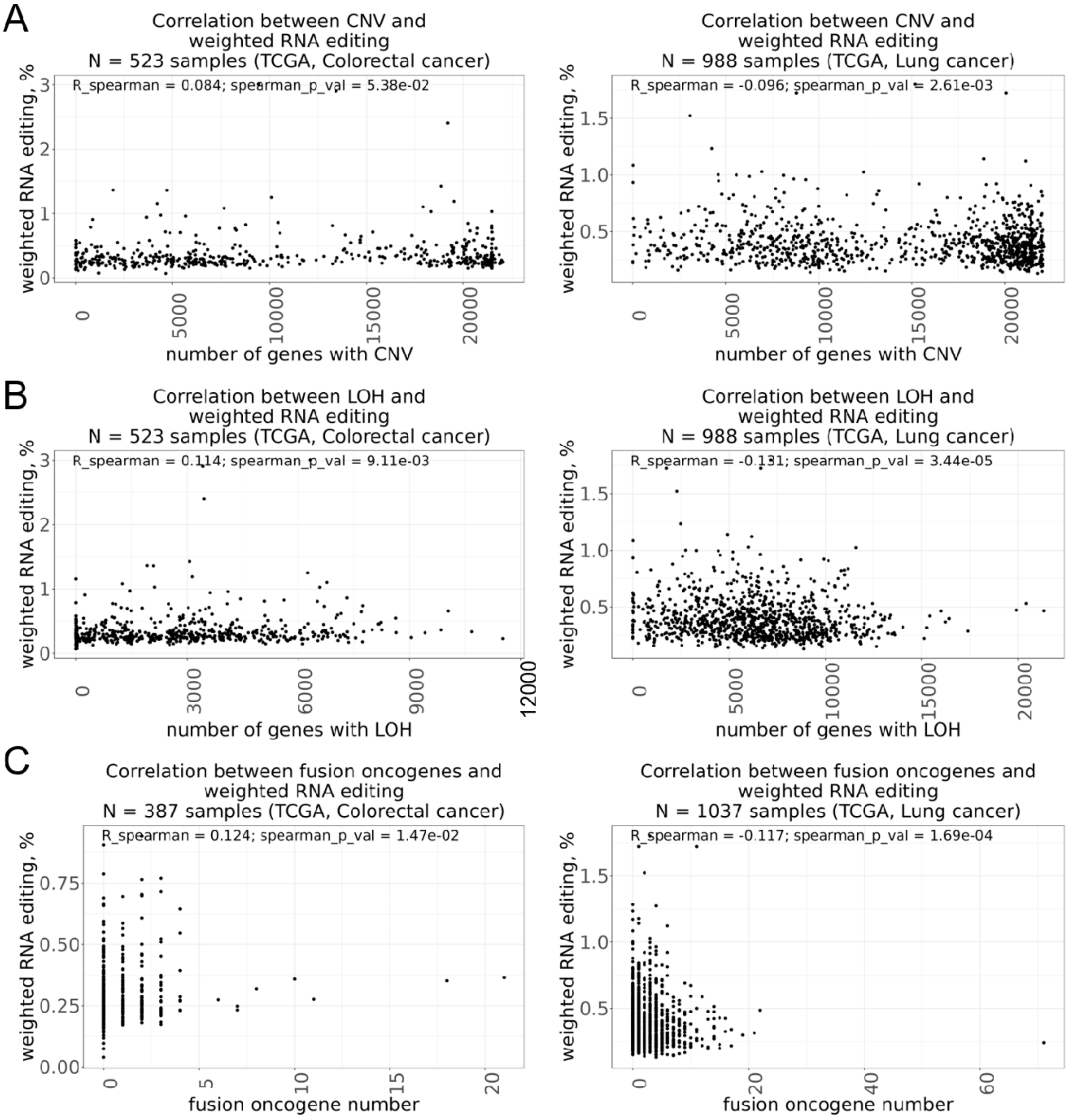

Figure S3. Spearman's rank correlation between the weighted RNA editing level and copy number variation (CNV) (A), loss of heterozygosity (LOH) (B), and fusion oncogene number (C) in TCGA cancer samples. Colorectal cancer samples are shown in the left panels, and lung cancer samples in the right panels.

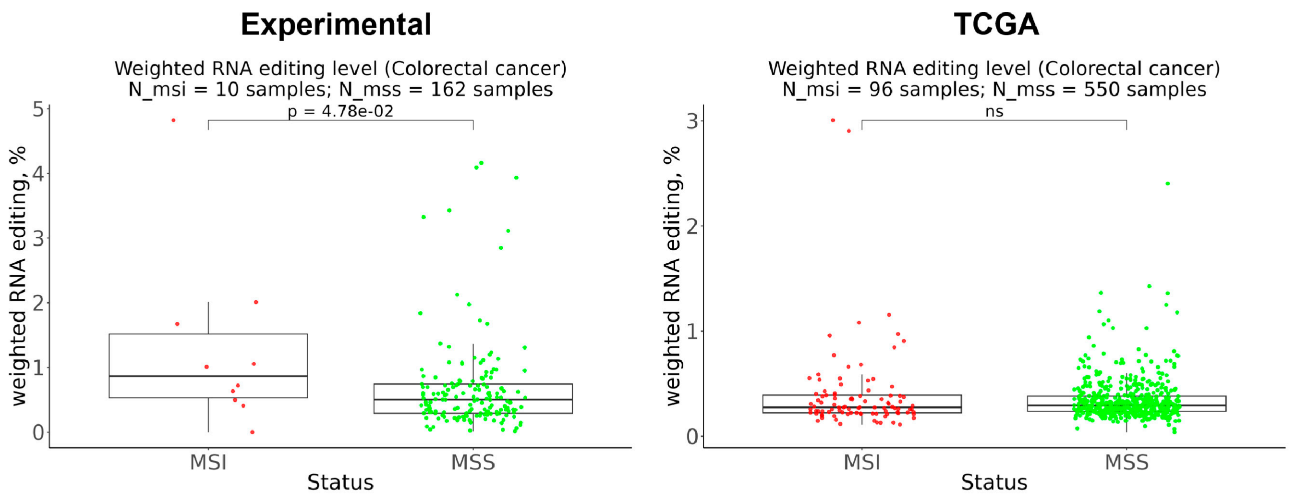

Figure S4. Differential analysis of ADAR-mediated RNA editing levels between colorectal cancer samples with known microsatellite instability status in the experimental (left) and TCGA (right) cohorts. MSI - microsatellite unstable, MSS - microsatellite stable. Mann-Whitney U test and Benjamini-Hochberg p-value correction (adjusted p-value < 0.05) were performed to assess statistical significance of the differences.
